# Acute lung injury induced by whole gastric fluid: hepatic acute phase response contributes to increase lung antiprotease protection

**DOI:** 10.1186/s12931-016-0379-7

**Published:** 2016-06-14

**Authors:** Pedro Ayala, Manuel Meneses, Pablo Olmos, Rebeca Montalva, Karla Droguett, Mariana Ríos, Gisella Borzone

**Affiliations:** Department of Respiratory Diseases and Medical Research Center, Faculty of Medicine, Pontificia Universidad Católica de Chile, Marcoleta 350, piso 1, Santiago, Chile; Department of Nutrition, Diabetes and Metabolism, Faculty of Medicine, Pontificia Universidad Católica de Chile, Santiago, Chile; Department of Physiology, Faculty of Biological Sciences, Pontificia Universidad Católica de Chile, Santiago, Chile; Instituto Nacional del Tórax, Santiago, Chile

**Keywords:** Aspiration, Liver acute phase response, Acute phase proteins, α1-antitrypsin, α2-macroglobulin, Alveolar-capillary barrier, Lung antiprotease defense

## Abstract

**Background:**

Gastric contents aspiration in humans is a risk factor for severe respiratory failure with elevated mortality. Although aspiration-induced local lung inflammation has been studied in animal models, little is known about extrapulmonary effects of aspiration. We investigated whether a single orotracheal instillation of whole gastric fluid elicits a liver acute phase response and if this response contributes to enrich the alveolar spaces with proteins having antiprotease activity.

**Methods:**

In anesthetized Sprague-Dawley rats receiving whole gastric fluid, we studied at different times after instillation (4 h −7 days): changes in blood cytokines and acute phase proteins (fibrinogen and the antiproteases alpha1-antitrypsin and alpha2-macroglobulin) as well as liver mRNA expression of the two antiproteases. The impact of the systemic changes on lung antiprotease defense was evaluated by measuring levels and bioactivity of antiproteases in broncho-alveolar lavage fluid (BALF). Markers of alveolar-capillary barrier derangement were also studied. Non-parametric ANOVA (Kruskall-Wallis) and linear regression analysis were used.

**Results:**

Severe peribronchiolar injury involving edema, intra-alveolar proteinaceous debris, hemorrhage and PMNn cell infiltration was seen in the first 24 h and later resolved. Despite a large increase in several lung cytokines, only IL-6 was found elevated in blood, preceding increased liver expression and blood concentration of both antiproteases. These changes, with an acute phase response profile, were significantly larger for alpha2-macroglobulin (40-fold increment in expression with 12-fold elevation in blood protein concentration) than for alpha1-antitrypsin (2–3 fold increment in expression with 0.5-fold elevation in blood protein concentration). Both the increment in capillary-alveolar antiprotease concentration gradient due to increased antiprotease liver synthesis and a timely-associated derangement of the alveolar-capillary barrier induced by aspiration, contributed a 58-fold and a 190-fold increase in BALF alpha1-antitrypsin and alpha2-macroglobulin levels respectively (*p* < 0.001).

**Conclusions:**

Gastric contents-induced acute lung injury elicits a liver acute phase response characterized by increased mRNA expression of antiproteases and elevation of blood antiprotease concentrations. Hepatic changes act in concert with derangement of the alveolar capillary barrier to enrich alveolar spaces with antiproteases. These findings may have significant implications decreasing protease burden, limiting injury in this and other models of acute lung injury and likely, in recurrent aspiration.

## Background

Gastric contents aspiration is defined as the inhalation of gastric material into the airways below the level of the vocal cords, with consequences that are variable, ranging from an almost asymptomatic episode to severe acute lung injury (ALI) with high mortality [1, 2]. In fact, gastric contents aspiration is a major direct cause of ALI. Although the true incidence of aspiration-induced lung injury is difficult to estimate considering that many aspiration events are silent, trauma and ICU patients with altered states of consciousness as well as patients requiring emergency anesthesia are at high risk of aspiration [2]. The deleterious effects of aspiration also depend on the terrain in which it occurs, as recently shown by Tsai et al., who found that aspiration of gastro-esophageal reflux increases the risk of intensive care unit admission and mechanical ventilation use among patients with chronic obstructive pulmonary disease [3].

The initial local lung effects of aspiration have been extensively studied in animal models [2, 4–15]. These effects include severe derangement of the alveolar capillary barrier with extravasation of plasma constituents from the vascular to the alveolar spaces, polymorphonuclear neutrophil recruitment and expression of pro-inflammatory mediators. Recently, we contributed to the understanding of resolution of the initial ALI induced by whole gastric juice in a rat model [5], showing that resolution involves an organizing pneumonia with granuloma formation, that later resolves.

However, very little is known about effects outside of the lung that are likely to occur in association with local lung effects and that may have important implications in the course of ALI and its prognosis.

Stimulated by a complex intercellular signaling system involving pro-inflammatory cytokines, the liver responds to inflammation localized in distant organs, with the acute phase response (APR) [16], a response that has been studied in rodents with a variety of stimuli such as LPS, turpentine administration and exposition to thermal injury [17, 18], all stimuli that do not affect the lung primarily. This response is characterized by significant changes in blood levels of acute phase proteins (APPs), such as C-reactive protein, clotting and complement proteins and proteinase inhibitors [16].

Very limited studies have investigated the APR in relation to inflammatory processes affecting the lung primarily [19, 20] but interestingly, these studies have not evaluated whether or not liver-synthesized lung antiproteases are involved.

We hypothesized that aspiration-induced lung inflammation elicits a hepatic APR. We also hypothesized that increased blood levels of antiproteases will be part of this response and will contribute to increase bronchoalveolar lavage fluid (BALF) levels of antiproteases.

The aim of our work was to investigate whether and to what extent aspiration of gastric fluid induces a hepatic acute phase response in a rat model of orotracheal instillation of whole gastric fluid, and if so, to determine the effects of this response on lung antiprotease levels. This model has been well characterized by our group according to ATS recommendations [21], and meets all the requirements for a model of ALI with severe histological and physiological changes, which resolve leaving normal lung architecture [5].

We found that the severe lung inflammatory reaction induced by orotracheal instillation of whole gastric fluid elicits significant systemic effects that result in up-regulation of important lung antiproteases, contributing to improve lung antiprotease defences, underscoring an important role of the liver promoting lung tissue protection in acute lung injury. We believe that understanding how the liver response integrates with the local lung inflammatory response to promote immunity and tissue protection during aspiration of gastric contents will help to identify new approaches to treat this condition.

## Methods

### Animal model of orotracheal instillation of rat whole gastric fluid

The study was performed according to a protocol submitted to and approved by the Animal Research Ethics Committee of the Pontificia Universidad Católica de Chile.

#### Gastric fluid pool

Gastric fluid was obtained through a gastrotomy from adult male Sprague-Dawley rats (300 ± 10 g) fasted overnight and anesthetized intra-peritoneally with xylazine-ketamine (5.1 and 55.1 mg x kg^−1^, respectively). Gastric fluid samples were pooled, filtered through a 100 μm mesh filter and kept at −80 °C. Animals were euthanized thereafter by exsanguination under anesthesia.

#### Orotracheal instillation

Under the same anesthetic protocol, another set of animals was orotracheally intubated with a 22G wire-fed catheter. Visualization of the glottis was achieved using a modified human otoscope (Welch Allyn®). A volume of gastric fluid (1.5 ml x kg^−1^, pH = 1.69) previously found by us to distribute evenly (data not shown) was instilled and animals allowed to recover spontaneously from anesthesia.

#### Study groups

Animals were studied at 4, 12, 24 h, and at days 4 and 7 after instillation (*n* = 10 per group). Animals without intervention served as controls (*n* = 10).

#### Sample collection

Blood samples from the *vena cava* and snap frozen liver samples were obtained through a laparotomy prior to opening the thorax. Lungs were excised *en bloc* and the left main bronchus cannulated for bronchoalveolar lavage. For each animal, three aliquots of 0.15 M saline (1 ml each) were instilled, immediately aspirated, pooled and stored at −80 °C. BALF percent recovery was 67 ± 7 %. The right middle lobe was excised to prepare 10 % tissue homogenates in phosphate buffer. The caudate lobe was used to obtain the wet-to-dry weight ratio. The right lower lobe was fixed at 20 cm H_2_O with 10 % buffered formaldehyde solution and paraffin-embedded for histologic studies. The right upper lobe was excised and frozen for biochemical and molecular analysis.

### Histologic evaluation

For each animal, four whole lobe longitudinal sections (5 μm) were obtained and stained with hematoxylin-eosin for analysis by light microscopy as in [5], following ATS recommendations [21].

### Markers of alveolar-capillary barrier derangement

- ***a****)****Total protein concentration in BALF*** was measured using the Bradford protein assay [22].
- ***b****)****Lung wet****-****to****-****dry weight ratio****:* The caudate lobe was removed and weighed immediately. After desiccation at 60 °C the lobe was weighed again to determine its wet-to-dry weight ratio.
- ***c****)* In a new set of animals (*n* = 5 per group), ***the Evans blue dye*** was utilized as a marker for derangement of the alveolar-capillary barrier [23]. Four hours prior to sacrifice, the Evans blue dye was injected via tail vein and its extravasation into BALF was quantified by spectrophotometry (Shimadzu, Japan).

### Levels of pro-inflammatory cytokines and IL-10

Serum, BALF and lung tissue homogenate levels of IL-6, IL-1β, TNF-α and IL-10 were measured in duplicate with commercially available enzyme-linked immunosorbent (ELISA) assay kits (Quantikine, R&D Systems, USA) according to the manufacturer’s instructions. Microplates were read using a microplate reader (BIO-TEK® Instruments, INC. Winooski, Vermont, USA).

### Levels of blood and BALF acute phase proteins

Changes in the level of three acute phase proteins were studied in plasma: fibrinogen, α1-AT and α2-MG, whereas changes in α1-AT and α2-MG were studied in BALF.

- ***a****)****Fibrinogen****:* Plasma fibrinogen levels were determined by the Von Clauss coagulation micro-method [24].
- ***b) α1-AT:*** Both abundance and bioactivity of this antiprotease were measured in plasma and BALF:
  - *Western blot analysis of α1-AT:* 30 μg of plasma or BALF proteins were separated by 10 % SDS-PAGE and immobilized onto NO_2_-cellulose membranes (0.45 μm pore; Thermo Scientific, Rockford, IL, USA). Membranes were probed with an anti-human α1-AT rabbit antibody (Sigma, Mississauga, ON, Canada) and then with a goat anti-rabbit peroxidase-conjugated antibody (Thermo Scientific, Rockford, IL, USA). α1-AT immunoreactivity was visualized by enhanced chemiluminescence (SuperSignal® Pico Chemiluminescent Substrate kit; Thermo Scientific, Rockford, IL, USA) and Kodak X-ray film. Densitometric analysis was performed using the Image J 5 Program. Equal loading was controlled by Ponceau staining.
  - *α1-AT bioactivity measured by the Elastase Inhibitory Capacity (EIC) colorimetric assay:* The inhibition that plasma and BALF samples exerted on the in vitro elastase-induced N-succinyl-Ala-Ala-Ala-p-nitroanilide (SLAPN; Sigma) hydrolysis test was studied [25, 26]. The rate of p-nitroanilide released at 25 °C was followed for 3.5 min at 405 nm wavelength using a microplate spectrophotometer reader (BIO-TEK® Instruments, Inc. Winooski, Vermont, USA). Serial dilutions were assayed in order to calculate the volume of plasma and BALF needed to produce 50 % inhibition of the in vitro elastase activity. Results were expressed as 1/dilution required for 50 % inhibition.
- ***c) α2-macroglobulin:*** Plasma and BALF levels were measured by ELISA (Abcam Inc. Cambridge, MA, USA) according to the manufacturer’s instructions. Microplates were read using a microplate reader (BIO-TEK® Instruments, INC. Winooski, Vermont, USA).

### mRNA expression of α1-AT and α2-MG in liver samples

- ***a) RNA extraction and reverse transcription:*** Total RNA was isolated from snap frozen liver samples using Total RNA Mini Kit Tissue (Geneaid Biotech Ltd., Taiwan) according to the manufacturer’s instructions and 2 μg of total RNA of each sample was treated with DNAse I (Amplification grade; Invitrogen, USA). The single-strand cDNA was synthesized using Superscript II Reverse transcriptase (Invitrogen, USA) in 20 μl total volume with Random Primer (Invitrogen, USA) according to manufacturer’s instructions.
- ***b) Semiquantitative Real-Time RT-PCR:*** The 7500 Fast Real-Time PCR System (Applied Biosystems) was used to quantify the relative gene expression of α1-AT and α2-MG in rat liver; GAPDH was chosen as the housekeeping gene. PCR was performed using sequence-specific primers for α1-AT, α2-MG and GAPDH (Table 1) in a reaction mix containing Fast SYBR Green PCR Master Mix (Invitrogen, USA). All real-time PCR assays were performed in duplicate.

**Table 1:** Primer sequences for RT-PCR and product sizes

Gene name	Primer sequence	Product size
α1-AT	sense: 5′- cca cgg tga agg tgc cca tga tga-3′	375 bp
	antisense: 5′- tca gca cag cct tat gca cag cct-3′	
α2-MG	sense:5′-atc tac atg gtg atg gtt cc-3′	209 bp
	antisense:5′- acc tca ttg gat gaa gac tg-3′	
GAPDH	sense: 5′-acc aca gtc cat gcc atc ac -3′	451 bp
	antisense: 5′-tcc acc acc ctg ttg ctg ta -3’	

The thermal cycling conditions included an initial activation step at 95 °C for 20 s, followed by 40 cycles at 95 °C for 3 s and 60 °C for 30 s with an ultimate melting cycle (95–60 °C). The annealing condition for GAPDH was 66 °C for 30 s. In order to verify the specificity of each product, amplified products were subjected to melting curve analysis as well as to electrophoresis.

Relative real-time RT-PCR quantitation was performed according to Livak and Schmittgen [27], using the comparative threshold cycle (C_T_) values [28]. The Delta- Delta C_T_ (ΔΔC_T_) was calculated according to Eq. 1:

1 $$ \Delta \Delta {\mathrm{C}}_{\mathrm{T}}={{\left({\mathrm{C}}_{\mathrm{T},\mathrm{Target}}\hbox{-} {\mathrm{C}}_{\mathrm{T},\mathrm{GAPDH}}\right)}_{\mathrm{time}}}_{\mathrm{X}}\hbox{-} {{\left({\mathrm{C}}_{\mathrm{T},\mathrm{Target}}\hbox{-} {\mathrm{C}}_{\mathrm{T},\mathrm{GAPDH}}\right)}_{\mathrm{time}}}_0 $$

where, (C_T,Target_ - C_T,GAPDH_) _*time* 0_ represents normalized expression in controls, and (C_T,Target_ - C_T,GAPDH_)_*time* X_ is the normalized expression at the different time points of the study. The relative fold expression (RFE) of the target genes was calculated as in Eq. 2:

2 $$ \mathrm{R}\mathrm{F}\mathrm{E} = {2^{\hbox{-}}}^{\Delta \Delta \mathrm{CT}} $$

### Statistical analysis

Non parametric analysis of variance (Kruskall-Wallis) and linear regression analysis were used [29]. Graph Pad Prism 5.0 software was utilized. A *p* value < 0.05 was considered statistically significant.

## Results

### Gastric fluid-induced acute lung injury and pro-inflammatory mediators

**Fig. 1:**
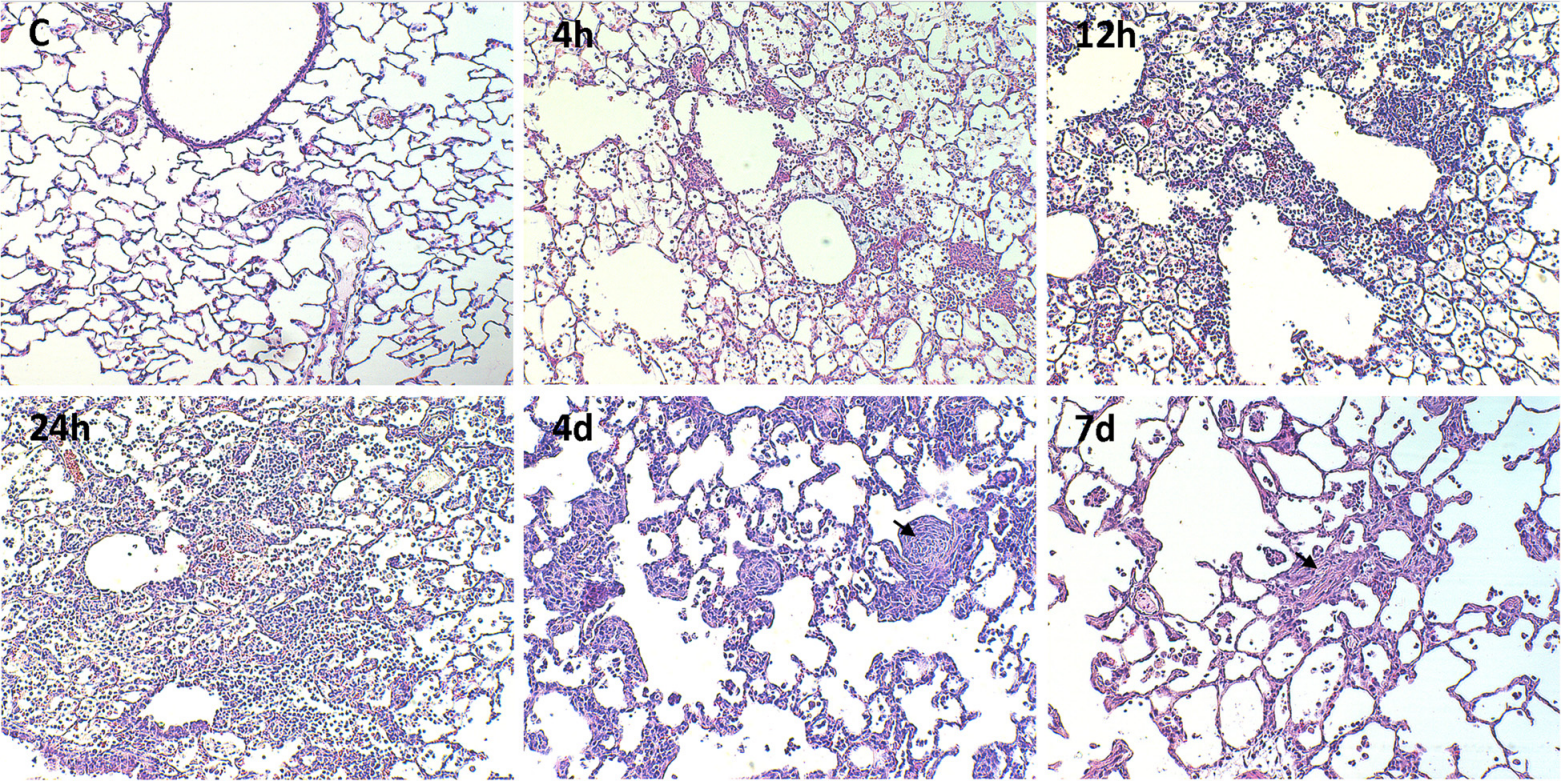
Lung inflammatory reaction after instillation of whole gastric fluid. Light microscopy (hematoxylin-eosin) of rat lung at 4, 12, and 24 h and, at days 4 and 7 after gastric fluid instillation. Intra-alveolar inflammatory and red blood cells along with proteinaceous material are seen at 4 h, with a peribronchiolar distribution. At 12 and 24 h, the inflammatory infiltrate is more intense with coalescence of affected areas and patchy consolidation. At days 4 and 7, intra-alveolar buds of granulation tissue (*arrows*) along with foreign-body giant cells, either isolated or forming granulomas are seen. Original magnification: 100×. C: control; d: days; h: hours

Histologic evaluation of the lung was performed at all-time points after instillation according to ATS recommendations [21]. Figure 1 shows results at several time points after instillation. At 4 h, increased alveolar thickening by interstitial edema and inflammatory cell infiltration, along with abundant protein-rich intra-alveolar exudate containing neutrophils and red blood cells were seen, adopting a peribronchiolar distribution. These changes became more intense at 12 and 24 h, with patchy consolidation due to coalescence of affected areas. At 4 and 7 days, foreign-body giant cells, either isolated or forming granulomas were frequently observed, along with intra-alveolar buds of granulation tissue. In the first 4 to 24 h, there was a large increase in inflammatory cells (more than 80 % PMNn cell predominance) whereas at days 4 and 7, mononuclear cells predominated [5].

**Fig. 2:**
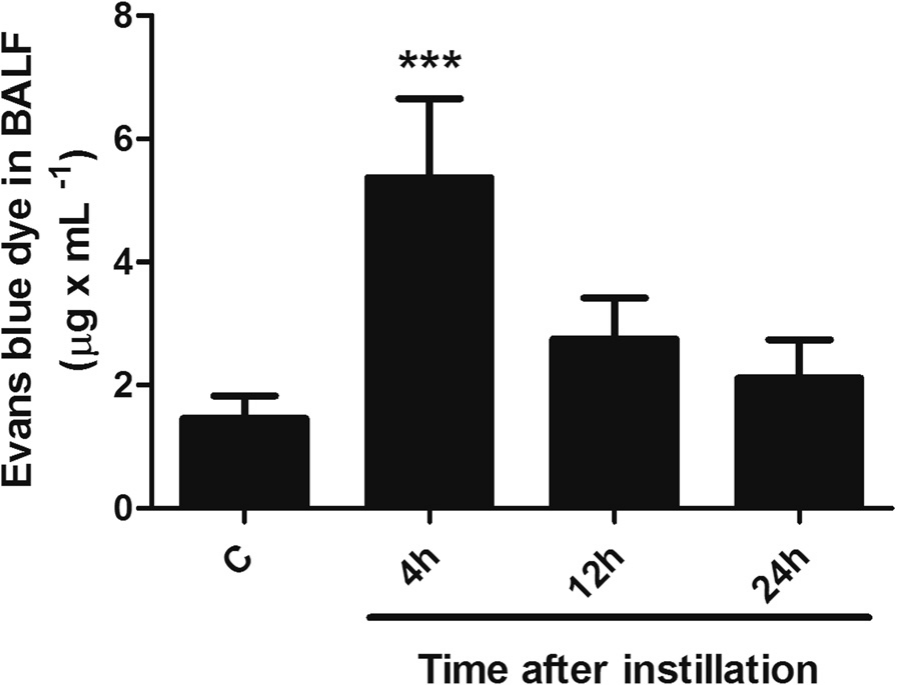
Evans blue dye extravasation into BALF after instillation of gastric fluid. A large extravasation of dye is seen at 4 h, with gradual return to control values thereafter. Values are means ± SD for *n* = 5 for each time point. ***: *p* < 0.001 with respect to control values. C: controls; h: hours

Markers of increased alveolar-capillary barrier permeability at different times after whole gastric fluid instillation are shown in Fig. 2 and Table 2. Using several markers of alveolar-capillary barrier permeability, the largest increase in permeability was seen at 4 h. Figure 2 shows that at 4 h there was a large extravasation of the intravenous Evans blue dye into BALF with gradual return to control values at 12–24 h, suggesting progressive restitution of barrier integrity. Non-significant changes in blood levels of Evans blue dye after IV injection were found. As seen in Table 2, lung wet-to-dry weight ratio showed a large but transient increase at 4 h (*p* < 0.001) with return to control values at 12 h. Table 2 also shows that BALF total protein concentration increased markedly (76 times the control value, *p* < 0.001) at 4 h and, although at a significantly lower level, it remained elevated at 12 and 24 h (*p* < 0.001).

**Table 2:** Markers of alveolar - capillary barrier derangement

	Control	4 h	12 h	24 h	4 d
Wet-to-dry weight ratio					
Mean	4,60	5,67 ***	4,71	4,56	4,80
S.D.	0,10	1,01	0,21	0,11	0,24
BALF total protein content					
Mean (mg x mL^−1^)	0,19	14,52 ***	10,78 ***	9,51 ***	0,88
S.D.	0,1	7,1	5,46	4,04	0,39

*h* hours, *d* days

***: *p* < 0,001 with respect to control values

**Fig. 3:**
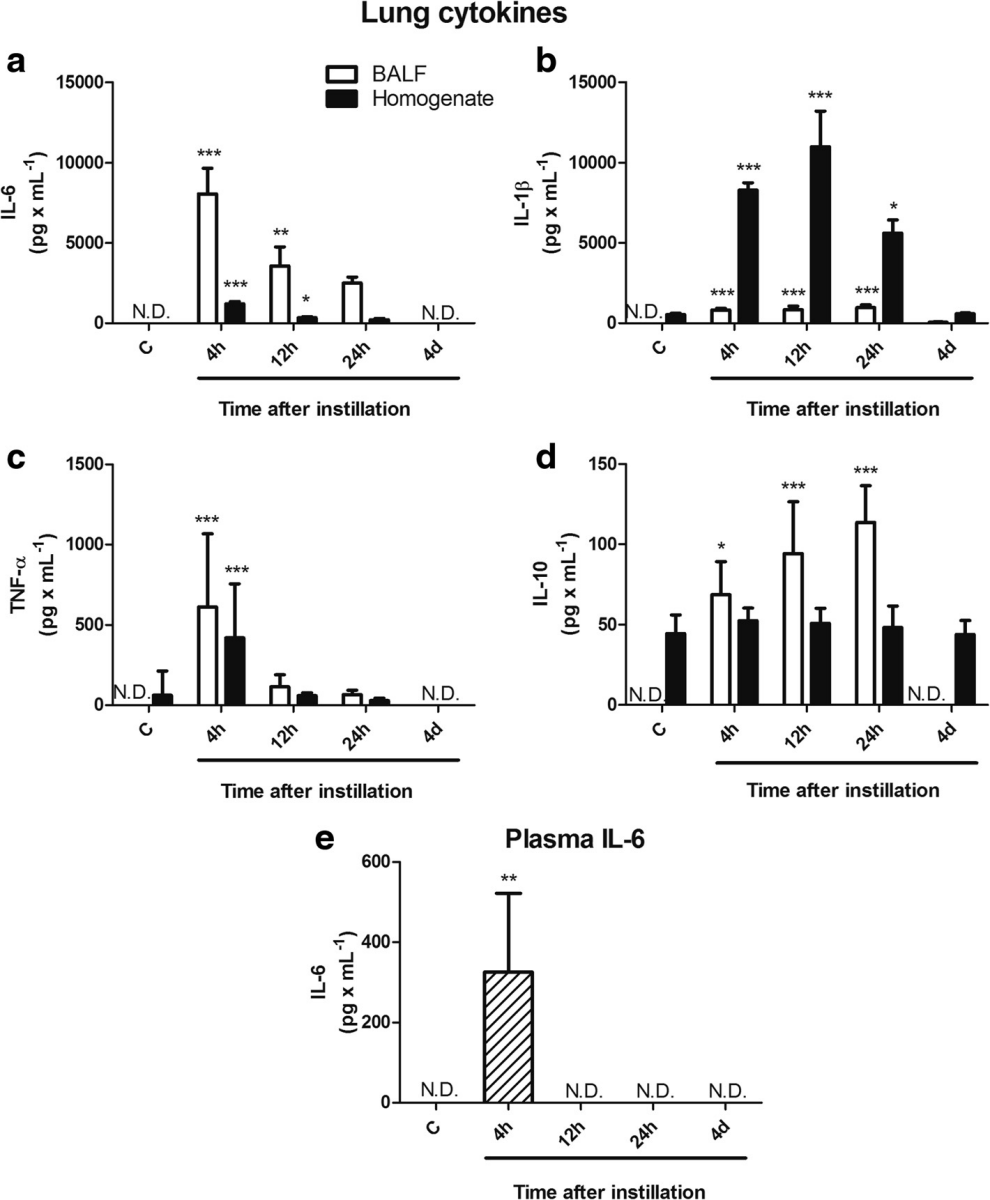
Pro-inflammatory cytokines and IL-10 levels in BALF, lung tissue homogenate and blood after instillation. **a** In the lung, IL-6 levels increased mainly in BALF, with a peak at 4 h and a progressive reduction to become non detectable at 4d. **b** IL-1β levels increased mainly in lung tissue homogenate with a peak level at 12 h and a significant reduction thereafter. **c** Levels of TNF-α showed a brief and moderate increment at 4 h both in BALF and lung tissue homogenate, with a significant reduction thereafter. **d** IL-10 levels in BALF showed a progressive increase between 4 h and 24 h and became non detectable at 4d, without changes in lung tissue homogenate. **e** In spite of the high levels of pro-inflammatory cytokines observed in the lung, only IL-6 was detected in plasma at 4 h after instillation. Open bars correspond to cytokine levels in BALF, solid bars correspond to cytokine levels in lung tissue homogenate and the hatched bar corresponds to IL-6 levels in plasma. Results are means ± SEM. *: *p* < 0.05; **: *p* < 0.01; ***: *p* < 0.001 with respect to control values. C: controls; h: hours; d: days; N.D.: non detectable

Changes in cytokines in BALF and lung tissue homogenates at different times after instillation are illustrated in Fig. 3. Pro-inflammatory cytokines IL-6, IL-1β and TNF-α were non-detected in control BALF and all showed an early increase in concentration after instillation, being mild for TNF-α and very large for IL-6 (Fig. 3a) and IL-1β (Fig. 3b). Whereas IL-6 increased mainly in BALF, IL-1β increased mainly in lung tissue homogenate, likely indicating differences in the cellular source of these cytokines. The highest levels of IL-6 were seen at 4 h (8,400 pg/ml, *p* < 0.001) with a progressive reduction later on. The highest levels of IL-1β were seen at 12 h (10,900 pg/ml, *p* < 0.001) with a significant reduction at 24 h (*p* < 0.01). Levels of TNF-α both in BALF and lung tissue homogenate showed a brief and moderate increase at 4 h (600 pg/ml, *p* < 0.001), with significant reduction thereafter (Fig. 3c). On the other hand, IL-10 levels showed a progressive increase between 4 and 24 h (*p* < 0.001) but only in BALF. In lung tissue homogenates, IL-10 remained detectable but unchanged throughout (Fig. 3d).

Changes in all measured cytokines in both BALF and lung tissue were transient and all the cytokines studied reached control levels at day 4.

In spite of the high levels of pro-inflammatory cytokines produced in the lung after instillation of gastric fluid, only IL-6 was detected transiently in plasma at 4 h (Fig. 3e).

### Acute phase proteins in blood

**Fig. 4:**
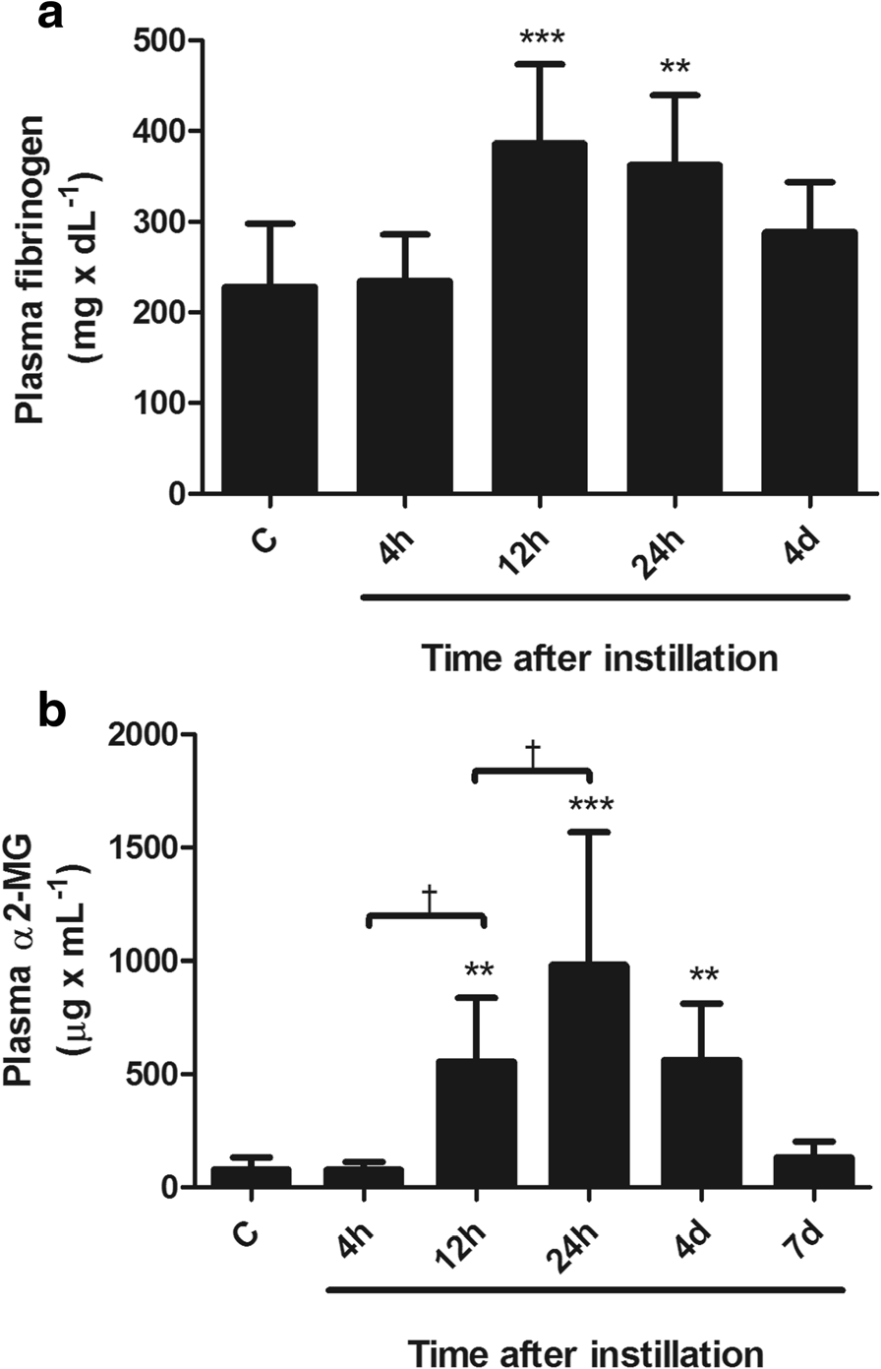
Changes in plasma fibrinogen and α2-MG concentration after instillation of whole gastric fluid. **a** Plasma levels of fibrinogen were not significantly different than in controls at 4 h. They increased at 12 h-24 h and returned to control values at 4d. **b** Plasma levels of α2-MG remained stable at 4 h, increased at 12 h, further increased reaching a peak at 24 h and decreased later on, but with still significantly high levels at 4d. Results are means ± 1SD; **: *p* < 0.01; ***: *p* < 0.001 with respect to control values; †: *p* < 0.05 with respect to the precedent time point value. C: controls; h: hours; d: days

**Fig. 5:**
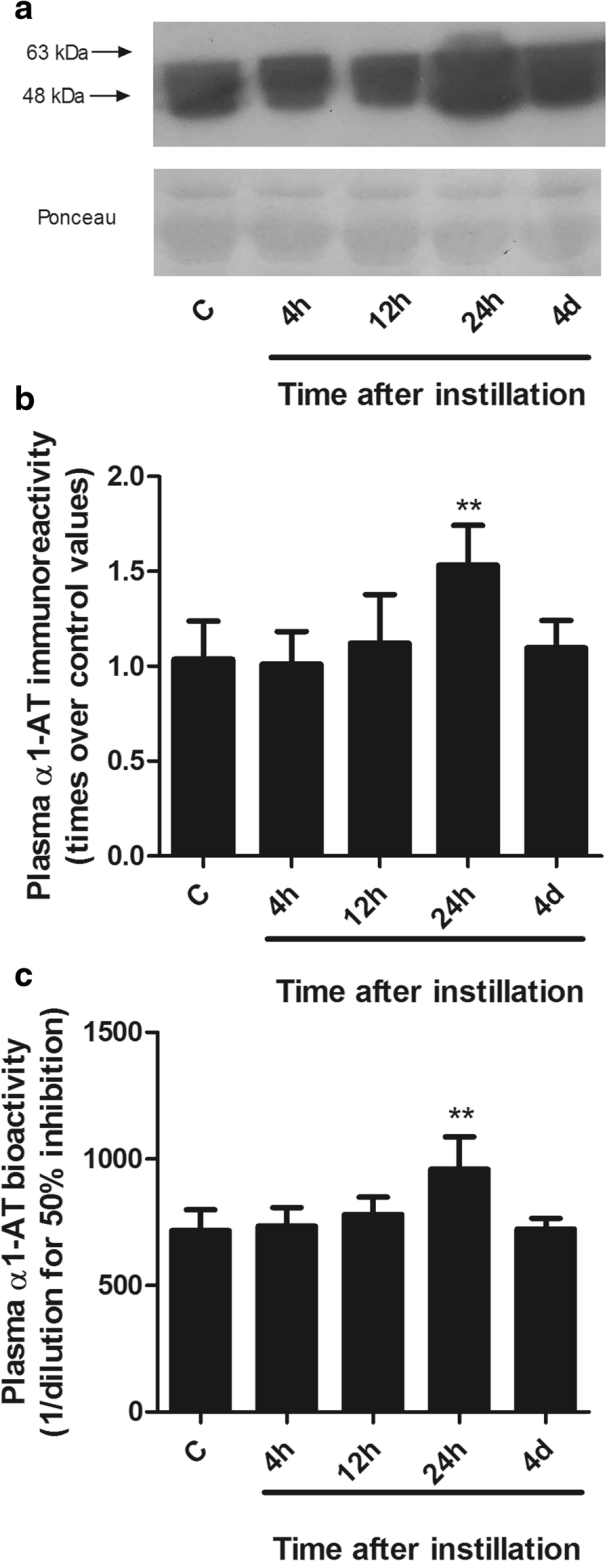
Changes in plasma α1-AT abundance and bioactivity after instillation of whole gastric fluid. **a** Representative Western blot of plasma samples illustrating the time course of changes in plasma α1-AT abundance. The ~55 kDa α1-AT immunoreactive band seen in the control sample shows a significant enlargement at 24 h. **b** Densitometric analysis of Western blots, normalized by their corresponding Ponceaus and expressed as times over control values (*n* = 5 for each time point). α1-AT immunoreactivity increased 1.5 times the control values at 24 h and was back to control levels at 4d. **c** Plasma α1-AT bioactivity increased 1.3 times with respect to control values at 24 h with return to control values at 4d. Bars are means ± 1SD; **: *p* < 0.01 with respect to control values. C: controls; h: hours; d: days

Changes in the levels of acute phase proteins in blood are shown in Figs. 4 and 5.

***Fibrinogen concentration in plasma*** was not increased at 4 h but showed a mean increase of 68 % at 12 and 24 h (*p* < 0.001), with return to control values at day 4 (Fig. 4a).

***α2-MG concentration in plasma*** was not increased at 4 h but showed a mean increase of 7.5 times above control values at 12 h (p < 0.01) and of 13.4 times at 24 h (p < 0.001), with significant reduction thereafter, to reach control values at day 7 (Fig. 4b).

***α1-AT abundance and bioactivity in plasma*** after instillation of whole gastric fluid are shown in Fig. 5. Figure 5a illustrates changes in plasma α1-AT abundance in a representative Western blot. The ~55 kDa α1-AT immunoreactive band that is seen in the control sample increases at 24 h after treatment and decreases at day 4. Figure 5b shows the results of the densitometric analysis of α1-AT Western blots, normalized by their corresponding Ponceaus and expressed as times over control values (*n* = 4 for each time point). α1-AT immunoreactivity increased 1.5 times at 24 h after instillation (*p* < 0.01) and was back to control values at day 4. Figure 5c shows that changes in plasma α1-AT bioactivity were similar to changes in immunoreactivity. At 24 h, α1-AT bioactivity increased significantly (1.3 times the control values (*p* < 0.01)), with return to control values at day 4.

The time course of changes in blood concentration of these three acute phase proteins, preceded by elevation of IL-6 levels in blood, was compatible with a rodent acute phase response profile [17].

### Gene expression of antiproteases α1-AT and α2-MG in the liver

**Fig. 6:**
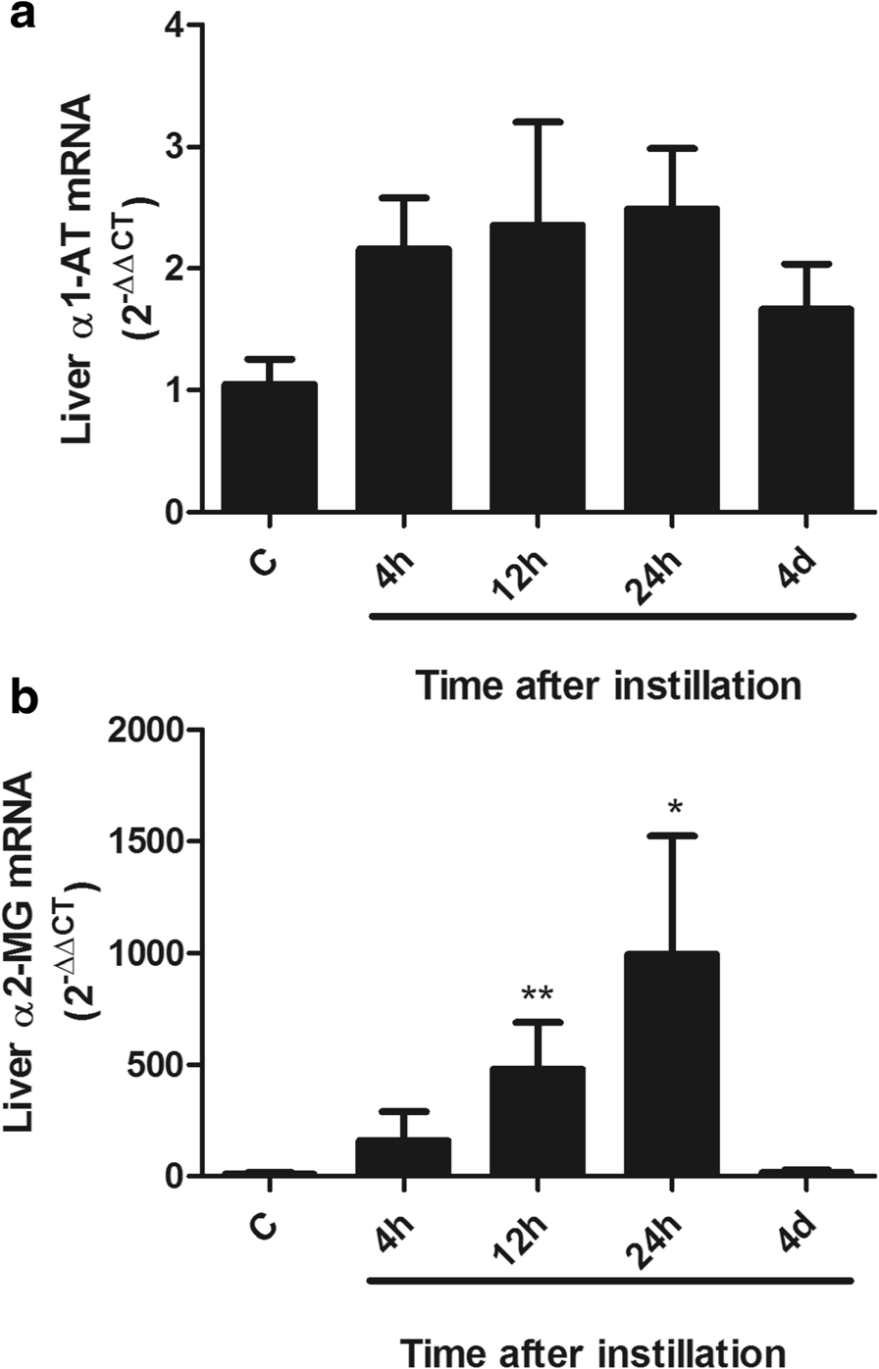
Changes in liver α1-AT and α2-MG gene expression after instillation of whole gastric fluid. **a** α1-AT gene expression: a non-significant two- to three-fold increase in liver α1-AT mRNA expression was seen between 4 h and 24 h. **b** α2-MG gene expression: a mean forty-fold increase at 4 h, 400-fold increase at 12 h and 900-fold increase at 24 h in α2-MG mRNA expression was seen. Results are means ± SEM; *: *p* < 0.05; **: *p* < 0.01 with respect to control values. C: controls; h: hours; d: days

In Fig. 6a, the two- to three-fold mean increase in α1-AT mRNA, that was seen at 4 h and persisted during the first 24 h, did not reach statistical significance. Nevertheless, this change in expression preceded small but significant changes in α1-AT protein abundance and bioactivity in blood with a liver acute phase response profile.

Figure 6b shows that changes in α2-MG mRNA expression were significantly larger than for α1-AT mRNA expression, with a forty-fold mean increase at 4 h, a 400-fold mean increase at 12 h (*p* < 0.01) and a 900-fold mean increase at 24 h (*p* < 0.05) and return to control values at day 4. Changes in hepatic α2-MG mRNA expression preceded changes in blood α2-MG protein concentration.

### Changes in lung antiprotease protection after instillation of gastric fluid

**Fig. 7:**
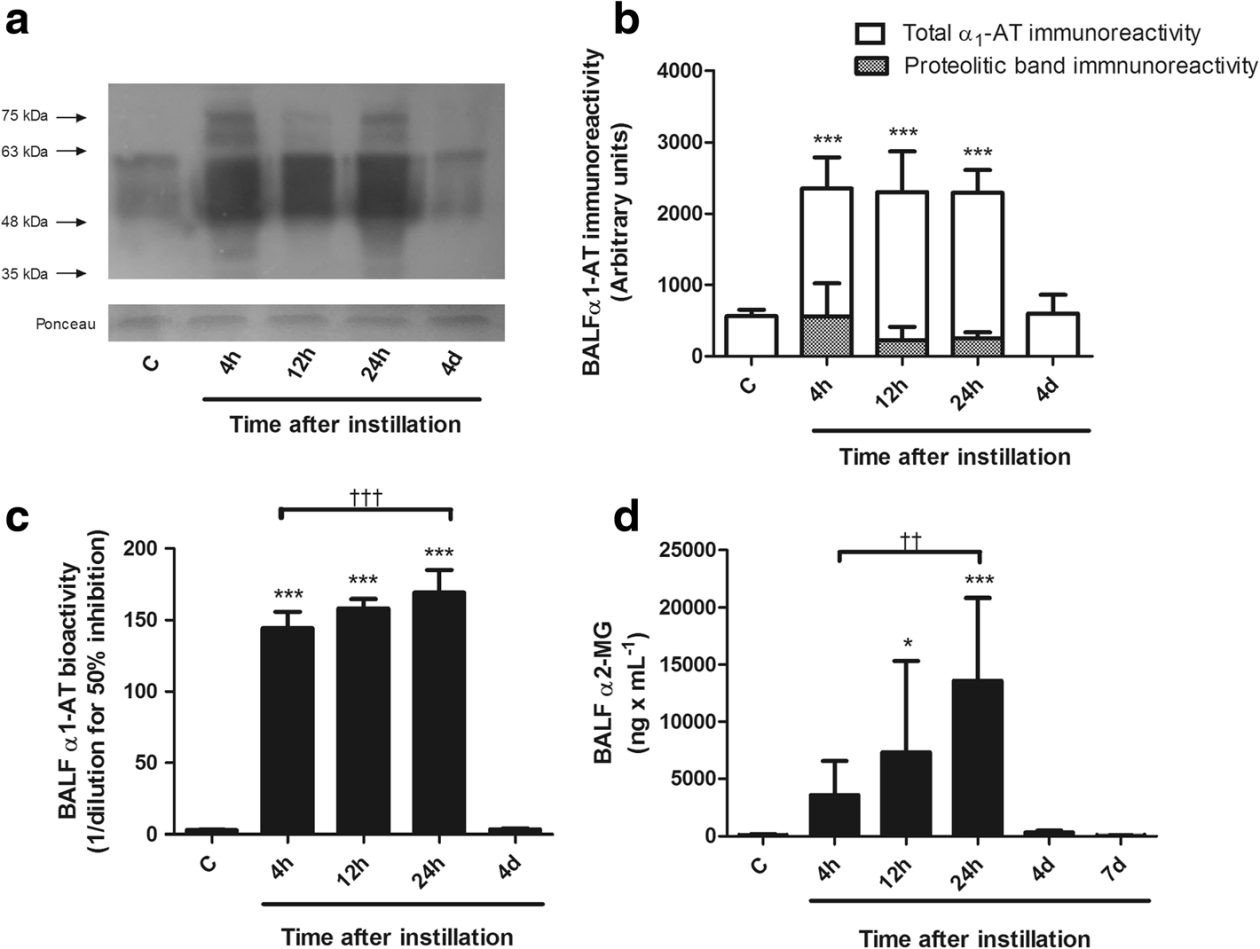
Changes in α1-AT and α2-MG in BALF after instillation of whole gastric fluid. **a** Representative Western blot illustrating changes in BALF α1-AT immunoreactivity. Two bands in the 55–60 kDa, corresponding to the native antiprotease are seen. After instillation, an approximately 88-kDa band corresponding to the [α_1_-AT-elastase] complex and a 45–50 kDa band, corresponding to the proteolytic fragment derived from the interaction between α_1_-AT and elastase are present at 4 h, 12 h and 24 h. In addition, the immunoreactivity of the 55–60 kDa band increases markedly at these time-points. Ponceau staining was used as a loading control. **b** Densitometric analysis of five independent Western blots. Total α_1_-AT immunoreactivity increased 4.2 times over control values at 4 h, remained elevated up to 24 h and returned to control values at 4d. Proteolytic band immunoreactivity was present only between 4 h and 24 h. Open bars correspond to the means ± 1SD of densitometric arbitrary units of total α_1_-AT immunoreactivity. Hatched bars correspond to immunoreactivity of the α_1_-AT proteolytic band. **c** Changes in α_1_-AT bioactivity in BALF. Elastase inhibitory capacity increased 49 times over control values at 4 h, with a further increase to 58 times the control values at 24 h and returned to control levels at day 4. **d** Changes in α2-MG concentration in BALF. α2-MG concentration increased significantly at 12 h, with a peak at 24 h. At 4d, α2-MG concentration was back to control values. Results are means ± 1SD; *: *p* < 0.05; ***: *p* < 0.001 with respect to control values; ††: *p* < 0.01; †††: *p* < 0.001 with respect to 4 h. C: controls; h: hours; d: days

Figure 7 illustrates lung consequences of local and hepatic changes induced by instillation of whole gastric fluid on lung antiproteases α1-AT and α2-MG.

#### BALF α1-AT abundance by Western blot analysis

Figure 7a shows a representative α1-AT Western blot of BALF samples. Control BALF shows two α1-AT immunoreactive bands in the ~55–60 kDa molecular size range, corresponding to the native α1-AT described in rodents [30]. After instillation, immunoreactivity of these bands increases and new bands are seen in the first 4–24 h: a ~88 kDa band corresponding to the [elastase-α1-AT] complex [31] and a ~45–50 kDa band corresponding to the α1-AT proteolytic fragment, derived from the interaction between elastase and α1-AT [31, 32]. At day 4, the immunoreactivity pattern of BALF α1-AT is similar to that in the control. Figure 7b shows the densitometric analysis of Western blots for α1-AT (*n* = 5 for each time point), with a mean 4.2 fold increase in α1-AT immunoreactivity that is evident at 4 h and remains elevated up to 24 h after instillation (*p* < 0.001).

#### BALF α1-AT bioactivity

Figure 7c shows changes in BALF α1-AT bioactivity after instillation. A significant increase in bioactivity was seen at 4 h (*p* < 0.001) with further increase later on. At 24 h α1-AT bioactivity was significantly higher than at 4 h (*p* < 0.001) and returned to control level at day 4.

#### BALF α2-MG concentration

Figure 7d shows changes in α2-MG concentration. This high molecular weight protein was found in very low concentration in control BALF. A progressive increase in concentration was seen in the first 24 h (*p* < 0.001), starting at 4 h. A mean 51-fold increase in concentration at 4 h (that did not reach statistical significance), 105-fold increase at 12 h and 194-fold increase at 24 h is shown, with return to control levels at day 4, despite still increased levels of α2-MG in plasma.

### Two-phase response in antiprotease enrichment of the alveolar spaces

As seen in Fig. 7, α1-AT bioactivity (7c) and α2-MG concentration (7d) in BALF at 4 h exhibit a similar large increment: a 40-fold and a 51-fold increase respectively in comparison to control values. These early changes in BALF occur without changes in blood levels of antiproteases as seen in Figs. 4b and 5. Instead, they occur in relation to the alveolar-capillary barrier derangement observed at 4 h, as illustrated by Evans blue dye extravasation and the significant increase in BALF total protein content and in lung tissue wet to dry weight ratio.

**Fig. 8:**
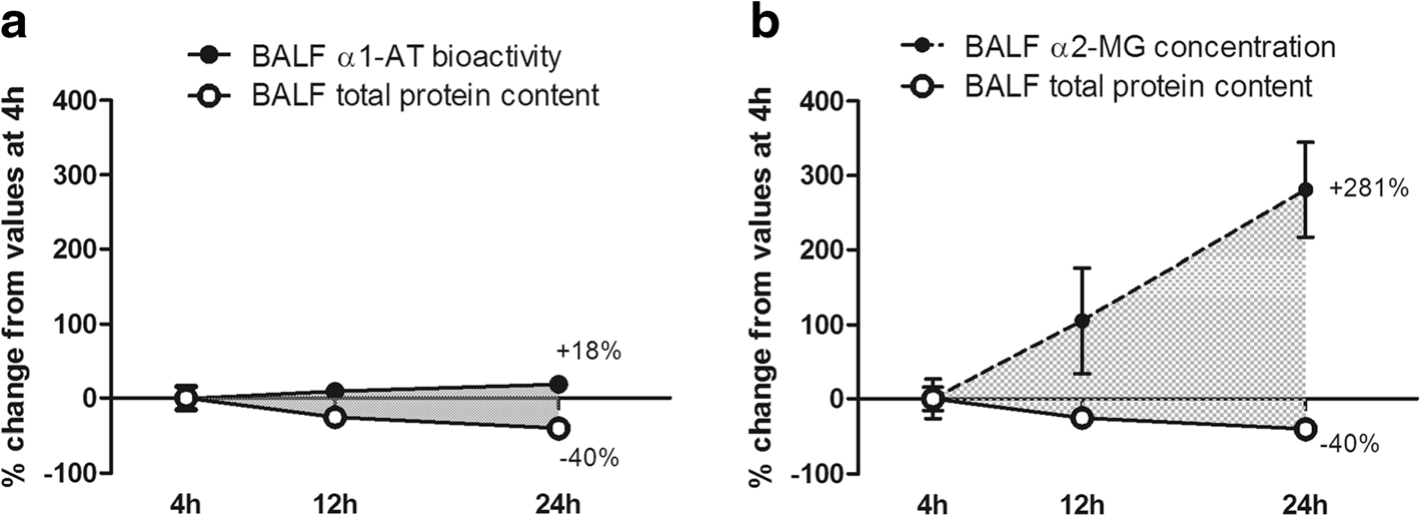
BALF antiprotease enrichment after the first 4 h, due to increased capillary-alveolar concentration gradient without further increase in permeability. **a** Changes in BALF α1-AT bioactivity and BALF total protein content between 4 h and 24 h after instillation. At 24 h, while total protein content falls 40 %, BALF α1-AT bioactivity increases 18 % above values observed at 4 h. **b** Changes in BALF α2-MG concentration and BALF total protein content between 4 h and 24 h after instillation. At 24 h, while total protein content falls 40 %, BALF α2-MG concentration increases 281 % above values observed at 4 h. h: hours

After the first 4 h, there is a further rise in the levels of both antiproteases in BALF, but with different slopes, as seen in Fig. 8. This figure summarizes the magnitude of changes in BALF antiproteases occurring in relation to their increased blood levels (as percent changes from values at 4 h). An extra 18 % increase in α1-AT bioactivity (Fig. 8a) and an extra 281 % increase in α2-MG concentration (Fig. 8b) were found in BALF. These BALF changes occur together with an increment in the levels of both antiproteases in blood (Figs. 4 and 5). During this period of time, there is no further derangement of the alveolar-capillary membrane. In fact, Evans blue dye extravasation returns to control values (Fig. 2), BALF total protein content decreases progressively up to 40 % and wet to dry weight ratio reaches control values at 12 h (Table 2). Thus, during this period in which there is no further increase in permeability, antiprotease enrichment of the alveolar spaces likely occur in relation to the increment in the antiprotease capillary-alveolar concentration gradient, due to increased blood levels of antiproteases.

**Fig. 9:**
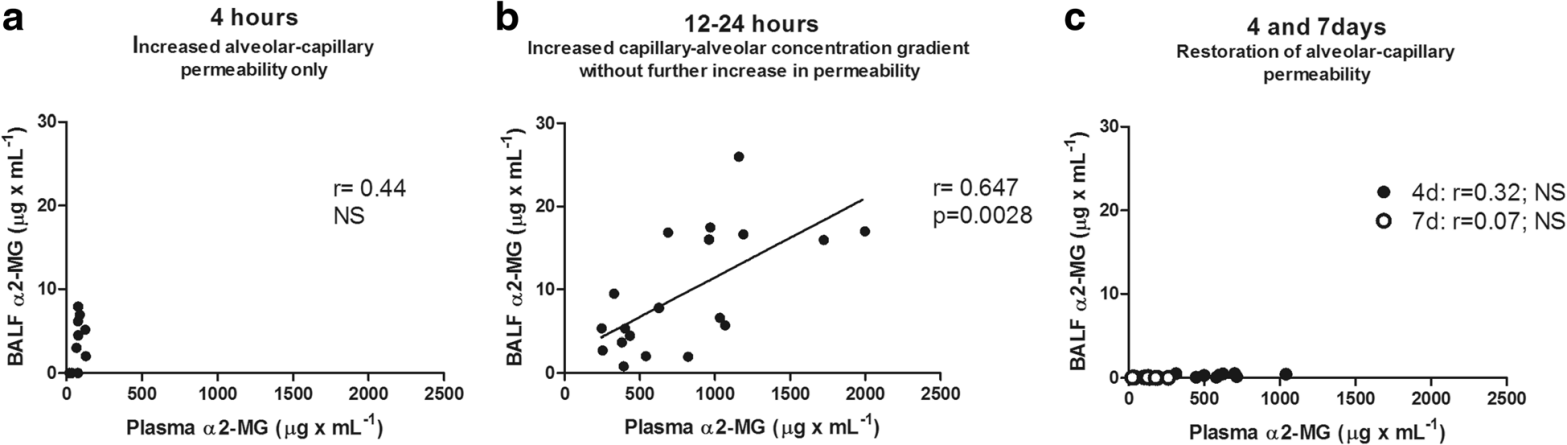
Relationship between plasma and BALF α2-MG concentrations.**a** Relationship between plasma and BALF α2-MG concentration at 4 h. Lack of correlation (*r* = 0.44; p N.S.) between plasma and BALF levels of α2-MG is seen. Increased BALF α2-MG concentration without an increase in plasma α2-MG concentration indicates loss of the protein size selectivity of the alveolar-capillary barrier. **b** Relationship between plasma and BALF α2-MG concentration at 12–24 h. A significant positive correlation (*r* = 0.647; *p* = 0.0028) between plasma and BALF levels of α2-MG at 12–24 h is seen, indicating that BALF levels of α2-MG during this period are determined by the increase in α2-MG capillary-alveolar concentration gradient, due to the liver acute phase response. **c** Relationship between plasma and BALF α2-MG concentration at 4 and 7d. Still elevated levels of α2-MG in blood at 4d (*solid circles*) do not result in elevated levels of α2-MG in BALF, reflecting restoration of the normal protein size selectivity of the alveolar-capillary barrier. At 7d (*open circles*), blood and BALF levels of α2-MG are back to control values. h: hours; d: days

Figure 9 shows the effects of the two processes that occur in the first hours after instillation and that determine largely increased levels of α2-MG in BALF: a) the alveolar-capillary barrier derangement and b) the increase in blood concentration of α2-MG. In Fig. 9a, α2-MG levels in BALF at 4 h do not correlate with α2-MG levels in blood, indicating that BALF levels of α2-MG are determined by derangement of the alveolar-capillary barrier that loses its protein size selectivity. In Fig. 9b, a significant positive correlation is seen between α2-MG levels in blood and α2-MG levels in BALF at 12–24 h. Data on plasma and BALF α2-MG concentration fit a lineal regression of the form *y* = 0.0095x + 1.941 (*r* = 0.64; *p* = 0.028). In this time period, markers of increased alveolar capillary barrier permeability progressively decrease, indicating that BALF levels of α2-MG are determined by the APR that increases the α2-MG concentration gradient. In Fig. 9c, still elevated levels of α2-MG in blood at day 4 do not result in elevated levels of α2-MG in BALF, reflecting restoration of the normal protein size selectivity of the alveolar-capillary barrier. Figure 9c also shows that at day 7, blood and BALF levels of α2-MG are back to control values.

## Discussion

Our results show for the first time, that a severe lung inflammatory reaction induced by orotracheal instillation of whole gastric fluid elicits a liver acute phase response, with elevation of acute phase proteins in the blood stream. The main lung antiproteases are part of this response, since mRNA transcription of α1-AT and mainly of α2-MG, is induced early in the liver, preceding elevation of the acute phase proteins in blood. Our results also show that the liver response contributes to the enrichment of the alveolar spaces with acute phase proteins that are important lung antiproteases. We identified a two-phase response in antiprotease enrichment of the alveolar spaces after aspiration, related to an early increase in alveolar-capillary barrier permeability and a later increment in capillary-alveolar antiprotease concentration gradient, due to increased blood concentration of antiproteases by increased liver synthesis.

### Biology of the acute phase response

The systemic inflammatory response is a collection of coordinated physiologic changes initiated during early stages of inflammation as part of the early innate defense, triggered by a variety of stimuli with the goal of achieving homeostasis. A prominent feature of this response is synthesis of several proteins mainly by the liver, under the control of cytokines originating at the site of injury [16], referred to as the liver acute phase response (APR). Changes in blood during the hepatic APR have been studied in rodents in response to stimuli not affecting the lung primarily, such as LPS and turpentine administration and exposition to thermal injury, and a number of APPs have been shown to participate. With these stimuli, the rodent APR starts a few hours after the insult, reaches a peak 24–48 h later and is back to baseline values at day 7 [17, 18, 33]. In general, it is assumed that elevation of APPs in blood have beneficial effects based largely on the known function of each of the individual proteins involved and on logical speculations as to how these might be useful in inflammation [16]. However, in the last few years, some negative implications of the hepatic APR have also been proposed, since the increase in blood of proteins with pro-coagulant effects such as fibrinogen, in relation to lung inflammation induced by urban air pollution and cigarette smoking, has been implicated in adverse cardiovascular effects [34]. In addition, this response has been implicated in the progression of lung damage in patients with COPD [35]. Unfortunately, studies relating cardiovascular morbidity and mortality during episodes of increased air pollution have focused only on C-reactive protein and APPs with pro-coagulant effects and have not evaluated the full spectrum of proteins that are known to be co-expressed, among them several antiproteases that can reach the lung improving its defense properties.

### Acute phase response elicited by gastric fluid-induced lung inflammation

Although the innate immune response to gastric contents aspiration has been extensively studied in the local lung environment, we have very little insights into extra-pulmonary effects elicited by aspiration that could have implications modifying lung inflammation and the prognosis of a single aspiration event. As a matter of fact, there is little information on the cascade of cytokines released in the lung during aspiration-induced ALI that could induce an APR [2, 5]. It has been proposed that the pattern of APPs produced in response to an injury and the cytokines involved in their regulation depends on the nature of the inflammatory stimulus [36]. Although it is known that IL-6, IL-1β and TNF-α modulate the synthesis of APPs in adult human hepatocytes, IL-6 is the major cytokine involved in the synthesis of the full spectrum of APPs [16, 33, 37]. IL-6 responsive regulatory elements have been found in the α1-AT gene that are responsible for both basal and induced expression of α1-AT in different human cell types and in rat hepatocytes [33, 38, 39]. On the other hand, it has been shown that IL-6 defective mice have a severely compromised APR to turpentine-induced tissue damage, as well as an impaired response following LPS injection [40, 41] and bacterial pneumonia [19]. In our model, IL-6 may play a role in the APR, since it was the only pro-inflammatory cytokine found to have increased levels in blood preceding changes in APPs, despite very high levels of other pro-inflammatory cytokines in the lung. It is also possible however, that other regulators could be involved in our model, since for instance in rats, CINC-1 the counterpart of human IL-8, has also been involved in APP production [42]. In our study, the high levels of IL-6 and TNF-α observed in BALF in comparison with lung tissue homogenate suggest that epithelial and alveolar mononuclear resident cells might be the source of these cytokines. In this regard, Fujii et al. have shown that the interaction between macrophages and epithelial cells has a synergistic effect on the production and release of mediators involved in the systemic inflammatory response [43]. With regard to the cytokine IL-10, we found that its concentration in BALF increases at the time pro-inflammatory cytokine levels decrease, in agreement with the known role of IL-10 limiting the cascade of pro inflammatory cytokines in lung inflammation [44].

In the literature, there is no information on the type and kinetics of APP production during gastric juice-induced ALI. We found a significant increase in the three APPs studied, with a time course of a rodent APR [17, 18, 33]. Additionally, the increment in α1-AT concentration in blood was associated with a proportional increase in its activity. The increment in blood levels of α1-AT and α2-MG was preceded by an increase in their mRNA expression in the liver, with the largest increase being that of α2-MG, which is considered to be the main acute phase antiprotease in the rat [17]. As a matter of fact, α1-AT, α2-MG and fibrinogen belong to the same group of APPs [16], responding to the same type of signal, involving activation of intracellular tyrosine kinase JAK and the acute-phase responsive factor, now called STAT3 [36, 45, 54].

On the basis of timing and magnitude of the changes observed in our model, we speculate that the increase in liver synthesis of fibrinogen, α1-AT and α2-MG could be mediated by the IL-6 signaling, that is known to participate in activation of the JAK/STAT3 cascade. Future studies using IL-6 inhibition are warranted to further evaluate the role of this cytokine in gastric juice-induced APR.

Although α1-AT is the main lung antiprotease in steady-state conditions, exhibiting a broad range of anti-inflammatory and immunoregulatory activities [37, 46], it is not the main acute phase antiprotease in the rat. It is produced primarily by the liver, with additional sources such as peripheral blood monocytes and alveolar macrophages contributing with a small fraction to total synthesis. Literature shows that similar to the liver, these additional sources are also known to respond to inflammatory stimuli with a small local APR, but their contribution to the increment in capillary-alveolar concentration gradient and to BALF APP levels is small [19, 47].

It has been shown that α1-AT is produced as part of the liver APR after turpentine injection in guinea pigs [48] and rats [17]. Support for a beneficial effect of α1-AT as part of the APR is provided by a study in a rat model of renal ischemia-reperfusion injury, in which the administration of α1-AT, at a dose that results in plasma levels similar to those observed during an APR, reduces inflammation, apoptotic activity and tissue damage [49]. Interestingly, we found complex formation between α1-AT and elastase in BALF between 4 and 24 h after instillation, providing evidence of the functionality of this antiprotease in our model.

With regard to α2-MG, it inhibits different types of proteases and is also a carrier and regulator of the function of several cytokines. Its large size prevents it from diffusing easily through the normal alveolar-capillary barrier and thus, it is found in very low concentration in normal alveolar spaces [46, 50]. It reaches the alveolar spaces in significant amounts whenever there is alveolar-capillary barrier derangement. Its role as a marker of alveolar-capillary barrier permeability has been studied in human acute respiratory distress syndrome [47]. In this condition, α2-MG has been found forming complexes with proteases and IL-8 [51]. Very little is known about its role as an acute phase protein in humans, however, it is recognized as the main acute phase antiprotease in the rat [17].

### Impact of the hepatic APR on lung antiprotease defense

Containing the lung inflammatory response induced by gastric juice is critically important in order to inhibit progression to a persistent systemic inflammatory response fueled by persistently increased cytokine production. In this regard, liver-newly synthetized antiproteases reaching the alveolar spaces may play an important role limiting lung inflammation. It has recently been shown that the APR has a role improving animal survival in experimental bacterial pneumonia [19, 52, 53] and although in these studies antiproteases were not evaluated, other APPs that were increased in blood were found increased in BALF [19]. On the other hand, research using APR-null mice in pneumonia has shown that in the absence of an APR, APPs do not increase in BALF and there is increased animal mortality [19, 54, 55]. Our results showed a highly significant enrichment in antiprotease content of the alveolar spaces, with a mean 58-fold increase in α1-AT activity and a mean 192-fold increase in α2-MG concentration in BALF. Given the fact that in our model, the acidic component of gastric contents triggers an early derangement of the alveolar-capillary barrier that precedes in several hours the increment in blood APP concentrations, we were able to show a two-phase response in the enrichment of the alveolar spaces with antiproteases.

### Two-phase response in the enrichment of the alveolar spaces with antiproteases

The first phase is characterized by an early and large increment in BALF antiprotease concentration and occurs prior to APP elevation in blood, thus not due to the APR, and instead due to derangement of the alveolar - capillary barrier that loses its protein size selectivity, facilitating the passage of both small (α1-AT) and large (α2-MG) molecular weight proteins to the alveolar spaces [50]. This phase contributes with a 40-fold increase in lung α1-AT bioactivity and a 51-fold increase in α2-MG concentration.

The second phase is contributed by the liver acute phase response that increases the capillary-alveolar concentration gradient for α1-AT and mainly for α2-MG. This phase provides an extra 18 % increase in BALF α1-AT bioactivity and an extra 281 % elevation in BALF α2-MG concentration (above the high levels provided by the first phase of antiprotease enrichment found at 4 h), in the context of significant reduction in total protein content in the same period of time. Interestingly, the antiprotease with the largest change in BALF in this phase is α2-MG that has also the largest change in blood concentration and in liver mRNA expression.

This two-phase response in lung antiprotease enrichment is likely to be unique to this model and not easy to detect in other models of ALI, in which alveolar-capillary barrier derangement is delayed by depending more on the effects of the inflammatory response than on the direct early effect of the acidic component of gastric juice.

It is also possible that a small part of the lung enrichment in antiproteases may come from alveolar mononuclear cells, that are known to respond to inflammatory stimuli with a low grade local lung APR [19, 47]. However, given the time-course of changes, this factor is unlikely to play a major role in our model, since antiprotease flooding occurs very early, prior to and during PMNn cell infiltration, and prior to mononuclear cell predominance, which occurs after day 4 [4, 5]. Furthermore it is accepted that the small amount of α1-AT released from monocytes serves more as a microenvironmental front line defense against proteases provided by the same monocytes [47].

On the basis of our results, local lung effects of gastric fluid damaging the alveolar-capillary barrier act in concert with lung inflammation-induced hepatic acute phase response to favour the arrival of new antiproteases to improve lung defense. Evidence of complex formation between α1-AT and elastase released from the inflammatory cells in the first 24 h provide support for the idea that antiprotease enrichment of the alveolar spaces plays a role limiting lung injury by proteases in this model. Protease inhibition may represent one of many mechanisms involved in the significant capacity of the lung to repair the severe ALI induced by gastric fluid recently reported by our group [5].

We postulate that antiprotease supplementation during the window of opportunity in which the hepatic APR co-exists with increased permeability, could be useful during ALI, in species without an increment in α1-AT and/or α2-MG in the liver acute phase response. In addition, it could be possible that either liver diseases or polymorphisms in the acute phase response genes may constitute susceptibility factors for lung or other tissue injuries. In this regard, increased lethality from lung damage has been shown by Borzio et al. [56] in patients with liver cirrhosis. On the other hand, a reduced APR was implicated in the increased lethality observed in experimental cirrhosis [57]. In the light of our results it is possible that antiproteases as part of APR could be involved in the results of the above mentioned studies.

In addition, since the APPs in blood remain elevated for several days, it is likely that they may impact lung response to repetitive events of aspiration. Future studies of this response with different lung inflammatory stimuli should consider the evaluation of the full range of proteins that are co-expressed, including the antiproteases, in order to have a better appreciation of the net effects of the liver APR in lung defense.

## Conclusions

Our results provide insights into the role of the liver in the pathogenesis of acute lung injury. A severe lung inflammatory reaction induced by gastric fluid elicits a liver acute phase response involving the main lung antiproteases. Liver changes act in concert with the derangement of the alveolar-capillary barrier to enrich the alveolar spaces with antiproteases. These results support the possibility of therapeutic interventions such as antiprotease supplementation during the window of opportunity in which the hepatic APR co-exists with increased permeability.
